# Characteristics of sodium and water retention in rats with nephrotic syndrome induced by puromycin aminonucleoside

**DOI:** 10.1186/s12882-023-03367-z

**Published:** 2023-10-25

**Authors:** Zaiping Xu, Yunlai Wang, Ye Feng, Mo Yang, Gaoxiang Shi, Zihua Xuan, Fan Xu

**Affiliations:** 1 School of Pharmacy, Anhui University of Chinese Medicine, Longzihu Road 350, Hefei, Anhui 230012 China; 2 Anhui Province Key Laboratory of Chinese Medicinal Formula, Hefei, Anhui China; 3 Institute for Pharmacodynamics and Safety Evaluation of Chinese Medicine, Anhui Academy of Chinese Medicine, Hefei, Anhui China; 4 Scientific Research and Technology Center, Anhui University of Chinese Medicine, Hefei, Anhui China; 5 School of Integrated Chinese and Western Medicine, Anhui University of Chinese Medicine, Hefei, Anhui China

**Keywords:** Nephrotic syndrome, Sodium and water retention, Urinary sodium excretion, Epithelial Na^+^ channel, Aquaporin 2

## Abstract

**Introduction:**

Nephrotic syndrome (NS) is characterized by renal sodium and water retention. The mechanisms are not fully elucidated.

**Methods:**

The NS rat model was established by single intraperitoneal injection of 100 mg/kg puromycin aminonucleoside (PAN). The plasma electrolyte level and urinary sodium excretion were monitored dynamically. The changes of some sodium transporters, including epithelial Na^+^ channel (ENaC), Na^+^/H^+^ exchanger 3 (NHE3), Na^+^-K^+^-2Cl^−^ cotransporter 2 (NKCC2) and Na^+^-Cl^−^ cotransporter (NCC) in renal cortex at different time points and the level of peripheral circulation factors were detected.

**Results:**

The urinary sodium excretion of the model group increased significantly on the first day, then decreased compared with the control group, and there was no significant difference between the model group and the control group on the 12th day. The changes of peripheral circulation factors were not obvious. Some sodium transporters in renal cortex increased in varying degrees, while NKCC2 decreased significantly compared with the control group.

**Conclusions:**

The occurrence of NS edema may not be related to the angiotensin system. The decrease of urinary sodium excretion is independent of the development of albuminuria. During the 18 days of observation, it can be divided into three stages: sodium retention, sodium compensation, and simple water retention. The mechanism is related to the increased expression of α-ENaC, γ-ENaC, NHE3 and NCC in a certain period of time, the compensatory decrease of NKCC2 expression and the continuous increase of aquaporin 2 (AQP2) expression.

**Supplementary Information:**

The online version contains supplementary material available at 10.1186/s12882-023-03367-z.

## Introduction

Nephrotic syndrome (NS) is a common clinical syndrome caused by glomerular diseases. Its typical manifestations are massive proteinuria, hypoproteinemia, severe edema and hyperlipidemia. Most scholars believe that the pathogenesis of NS is mainly related to immune disorders, abnormal genetic structure of podocytes and systemic circulatory factors [1, 2]. Although edema is not the core laboratory diagnostic criteria of NS, it is the most common symptom of NS. In the early stage, local edema such as eyelids will occur, and systemic edema such as pleural effusion and ascites may occur in the later stage. Diuretics are often used to treat edema clinically, but they are prone to the risk of diuretic resistance and hypovolemic shock [3]. It is speculated that drugs may not effectively target the mechanism of the disease. Therefore, a comprehensive understanding of the mechanism of edema is helpful for the treatment of clinical patients.

Rats induced by adriamycin (ADR) and puromycin aminonucleoside (PAN) are currently recognized as animal models for NS [4]. ADR can change the glycoprotein metabolism of glomerular epithelial cells by inducing lipid peroxidation of glomerular epithelial cells, destroy the structure and function of glomerular filtration membrane, and eventually lead to membrane filtration barrier lesions and cause proteinuria [5]. Because ADR has a wide range of injurious effects, in addition to glomerular podocytes, it also involves other tissues, resulting in significant extrarenal effects. PAN is an analogue of puromycin antibiotics, which directly damages DNA through reactive oxygen species, resulting in podocyte foam and foot process fusion, and changes the morphology and anion distribution of glomerular epithelial cells, and has selective renal injury [6, 7]. Although it has been reported in the literature that the reduction of sodium excretion fraction was observed in these two animal models, however, there are also experimental data with opposite results: no significant changes in urinary sodium excretion were observed in NS rats induced by ADR [8]. In this study, the urinary sodium excretion of ADR-induced NS rats changed dynamically, and the decrease of urinary sodium excretion was only detected at a few time points. Our previous results showed that the urinary sodium excretion of rats increased significantly at the end of 6 weeks after ADR administration, but the ascites content of rats was not obvious, and only some rats had toe swelling [9, 10]. Although there is no dynamic observation of urinary sodium excretion in our experiment, combined with the above ADR-NS rat results, we speculate that the urinary sodium excretion in ADR-NS rat model is unstable.

By comparing the urinary sodium excretion of PAN-NS rats in the literature, we found that at the end of the experimental period (day 12 or day 14), the urinary sodium excretion increased from the lowest value on day 6 to a normal or higher level, basically reaching a positive balance of urinary sodium, but there was still edema [11, 12]. Consistent with the results of experimental animals, patients with NS edema gradually recovered from sodium retention to sodium balance with the disease process, and the degree of edema was also gradually decreased [13, 14]. The rebalancing of sodium reveals the body’s ability to self-regulate, but what substances dominate this change and how do they cause it? That sparked our interest.

Therefore, we used the method of dynamic observation of urinary sodium excretion and water metabolism in NS rats to fully grasp the pathogenesis of NS edema. In this study, we established PAN-NS edema model, collected plasma, urine and tissue samples regularly, and dynamically observed the changes of sodium and water retention related indexes and peripheral circulation factors. This study deepens the understanding of the mechanism of edema, and provides theoretical support for the prevention and treatment of NS edema.

## Materials and methods

The main reagents are described where they are used.

### Animal models and experimental protocols

64 male SD rats, SPF grade, weight 200 ± 20 g, purchased from Shandong Experimental Animal Center, certificate number: SCXK (Lu) 20190003. All animal experiments were reviewed and approved by the Experimental Animal Ethics Committee of Anhui University of Chinese Medicine, Hefei, Anhui (Approval Number: AHUCM-rats-2021068). The rats (4 rats in the model group and 4 rats in the pair-fed control group at each time point) were housed in individual metabolic cages under circadian light conditions 3 days before the start of the experiment. NS rats were established by a single intraperitoneal injection of 100 mg/kg PAN (lot: 80335; MedChemExpress, Shanghai, China). NS model rats were given free access to water, food, and a standard diet containing 0.18% sodium during the experimental period. To achieve the same daily sodium intake between groups, pair-fed control group were given ad libitum water and an average daily food intake of model groups [15]. Every morning at 8:00 am, 24-hour urine samples were collected in centrifuge tubes filled with liquid paraffin and the urine volumes were determined. The daily food intake of the rats was measured to evaluate the balance of urinary sodium, and the dietary intake of the rats in the model groups were ascertained to determine the dietary intake of the pair-fed control groups. Rats were anesthetized with phenobarbital sodium (50 mg/kg bw, ip) at day 1, 2, 3, 6, 9, 12, 15, and 18, blood and tissue samples were collected after complete anesthesia.

### Biochemical indicators and urine sodium excretion detection

The abdominal cavity was opened after anesthetized rats at different time points, and the volume of ascites was obtained by infiltrating quantitative filter paper and weighing. Plasma electrolyte levels were detected by automatic biochemical analyzer (Shimadzu, Kyoto, Japan). The 24-hour urine of rats in each group was collected at different time points every day, and the urine electrolytes were detected by automatic biochemical analyzer. Urinary protein concentration was detected by BCA method (BCA protein quantitative kit, lot: 0717A19; Leagene, Beijing, China), and urinary creatinine content was detected by deproteinization end point colorimetric method (Creatinine assay kit, lot: 1026A20; Leagene, Beijing, China). The daily urinary sodium balance was calculated as the difference between 24-hour sodium intake and urinary sodium excretion. Sodium excretion is expressed as the ratio of urinary sodium concentration to urinary creatinine concentration in order to avoid errors caused by changes in urinary sodium concentration due to dilution of urine samples and urine collection. Similarly, urinary protein excretion is expressed as the ratio of urinary protein content to urinary creatinine concentration.

### Body fluid content analysis

The body composition data of rats measured by ImpediVET™ Bioimpedance Spectroscopy device (ImpediMed Limited, Brisbane, Australia) mainly include total body water (TBW), extracellular fluid (ECF), Intracellular fluid (ICF), fat mass, fat-removed mass and body mass index, etc. TBW, ECF, and ICF can directly reflect the water content and distribution of the body [16]. Values were calculated as the average of three consecutive tests.

### Radioimmunoassay

Plasma processing: 3 mL of plasma was added to a centrifuge tube containing EDTA anticoagulant (30 µL 0.3 M EDTA, 15 µL 0.3 M dimercaptopropanol, 30 µL 0.35 M 8-hydroxyquinoline sulfate), shaken well, and centrifuged at 3500 rpm for 15 min at 4 °C. Supernatants were collected and stored frozen at -20 °C.

Renal tissue homogenate: Took 100 mg of tissue samples containing renal cortex and medulla, added 900 µL of normal saline, and added EDTA, dimercaprol and 8-hydroxyquinoline sulfate in the corresponding proportion according to the instructions, homogenated on ice, centrifuged at 4 °C, and took the supernatant for testing.

The concentration of Angiotensin II (Ang II), vasopressin (AVP) and renin activity in the samples were detected using a Gamma radioimmunoassay counter (Xi’an Nuclear Instrument Factory, Xi’an, China) according to the operating instructions of the radioimmunoassay kit (Ang II and Ang I radioimmunoassay kit, both lots are 191220, Beijing North Institute, Beijing, China; AVP radioimmunoassay kit, lot: 194604, DIA Source, Louvain-la-Neuve, Belgium).

### Immunohistochemistry and immunofluorescence staining

Immunohistochemical staining: The kidney tissue in coronal section was immersed in 4% paraformaldehyde solution for 24-hour. After dehydration, embedding, sectioning, dewaxing and washing, the sections were incubated with primary antibody at 4 °C overnight. The next day, sections were washed and incubated with secondary antibodies for 30 min, followed by 3,3 ′-diaminobenzidine (DAB) and hematoxylin staining, respectively. The sections were then examined and photographed using fluorescence microscopy (Olympus, Tokyo, Japan).

Immunofluorescence staining: After staining, sectioning, deparaffinization, and washing, the sections were incubated with primary antibody overnight at 4 °C. Then, the samples were washed three times with PBS, incubated with secondary antibody for 50 min, and then counterstained with DAPI at 25 °C. Photographs were collected by fluorescence microscopy.

### Western blot assay

Kidney tissue was lysed using RIPA tissue lysis buffer and measured for protein concentrations with BCA analysis. After electrophoresis, PVDF transfer and blocking, the blots were incubated overnight with primary antibodies against γ-epithelial Na^+^ channel (γ-ENaC, Abcam, Cambridge, UK), α-epithelial Na^+^ channel (α-ENaC, StressMarq Biosciences, Victoria, Canada), Na^+^-Cl^−^ cotransporter (NCC, Abcam, Cambridge, UK), Na^+^-K^+^-2Cl^−^ cotransporter 2 (NKCC2, StressMarq Biosciences, Victoria, Canada), Na^+^/H^+^ exchanger 3 (NHE3, Novus Biologicals, Colorado, USA), and Aquaporin 2(AQP2, Santa Cruz Biotechnology, Texas, USA) at 4 °C.Then, washed three times for 5 min. The blots were incubated with the secondary antibody (1:10000) at room temperature for 2 h and washed three times for 5 min. At last, the blots were observed by ECL and the protein bands were measured using Image J.

### Statistical analysis

All experimental results are expressed as mean ± SE. Statistical analysis was performed using SPSS 22.0 statistical software. The data were compared by two-way ANOVA followed by Student’s t-test for unpaired comparisons. *P* < 0.05 was considered a statistically significant difference, while *P* < 0.01 was considered to indicate extremely significant differences.

## Results

### Biochemical indicators and urinary sodium excretion

**Fig. 1:**
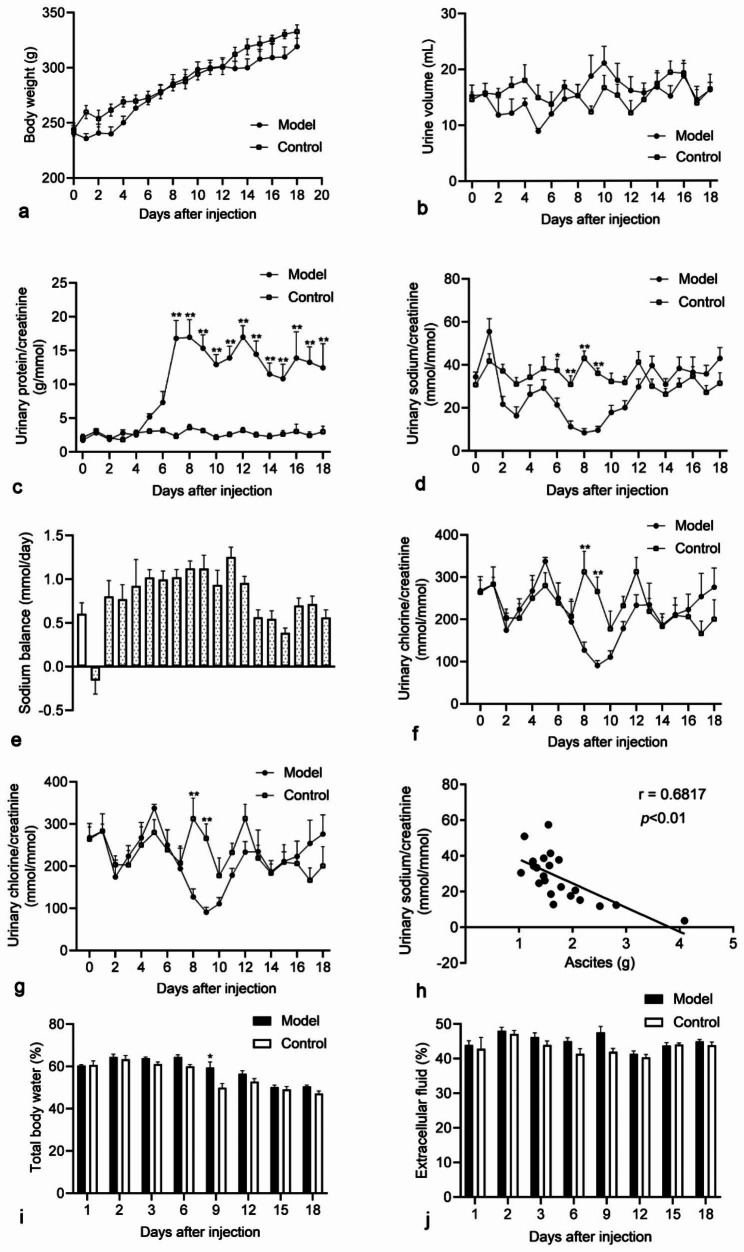
Changes of sodium and water retention indexes in PAN and control rats throughout the study. **a** The time courses of body weight. **b** The time courses of urine volume. **c** The time courses of proteinuria. **d** The time courses of urinary sodium. **e** sodium balance curve of model group. **f** The time courses of urinary chlorine. **g** The time courses of ascites. **h** Relationship between urinary sodium and ascite in NS rats. **i** Total body water in rats. **j** Extracellular fluid in rats. The values are mean + SE from four to six animals. * indicates a statistical difference of *P* < 0.05 between the model group and the control group, while ** indicates a statistical difference of *P* < 0.01 between the two groups

During the whole experimental period, the body weight of the control group and the model group increased steadily without difference (Fig. 1a). There was no difference in 24-hour urine volume between the control group and the model group (Fig. 1b). Proteinuria increased significantly on day 7 after PAN modeling (16.80 ± 2.65 vs. 2.31 ± 0.46 g/mmol creatinine, *p* < 0.01) and peaked on day 8 (16.96 ± 2.61 vs. 3.60 ± 0.44 g/mmol creatinine, *p* < 0.01), remained at high levels until day 18 (Fig. 1c).

Urinary sodium excretion showed a transient increase on the first day after PAN injection (55.51 ± 5.96 vs. 34.41 ± 2.25 mmol/mmol creatinine, *p* < 0.05, Fig. 1d). It was also increased in control rats receiving equal amounts of saline, but this increase was not significantly different (41.76 ± 3.41 vs. 30.80 ± 4.41 mmol/mmol creatinine, *p* > 0.05). Urinary sodium excretion subsequently decreased to moderate levels (21.71 ± 3.61 to 16.38 ± 4.11 mmol/mmol creatinine) on days 2 and 3, and reached very low levels (11.25 ± 2.65 to 8.46 ± 1.83 mmol/mmol creatinine) on days 7 to 9. Higher urinary sodium excretion was still observed in pair-fed controls, so there was no association between reduced urinary sodium excretion and food intake in PAN-NS rats. The decrease in urinary sodium excretion continued until day 12 and then increased to a range slightly above basal level. In the model group, urinary sodium was in positive equilibrium at all time points except for day 2 when urinary sodium excretion increased significantly, and excretion reached negative equilibrium (intake was less than excretion) (Fig. 1e). Combined with the urinary protein data, it can be seen that urinary protein began to appear on the 3rd day after the reduction of urinary sodium excretion, indicating that the appearance of proteinuria does not affect the process of urinary sodium excretion, suggesting that the reduction of urinary sodium excretion may not be related to the development of proteinuria. Urinary chlorine excretion decreased gradually on the fifth day after PAN modeling and was significantly higher than that of the control group on the 8th day (127.14 ± 19.06 vs. 312.60 ± 48.68 mmol/mmol creatinine, *p* < 0.01), reached the lowest value on the 9th day (90.96 ± 11.42 vs. 266.00 ± 34.05 mmol/mmol creatinine, *p* < 0.01), and then gradually increased (Fig. 1f).The results of ascites collected by the filter paper method showed that compared with the pair-fed control group, the rats in the model group had obvious ascites on the 6th, 9th, and 12th days (*p* < 0.01). Day 9 had the most ascites (2.89 ± 0.42 vs. 0.90 ± 0.05 g, *p* < 0.01, Fig. 1g). The results of Pearson correlation analysis showed that urinary sodium excretion was negatively correlated with ascites (Fig. 1h).

Rat plasma was collected at different time points to detect plasma electrolyte levels. Compared with the pair-fed control group, there was no significant difference in plasma sodium and plasma chloride content in the model group, and the plasma potassium concentration only increased significantly on the first day (5.15 ± 0.07 vs. 4.06 ± 0.12 mmol/L, *p* < 0.01), there was no difference at other time points (**Supplemental Fig. 1**), indicating that PAN-NS rats did not have significant hyponatremia during this observation period.

### Body fluid content of rats

The direct indicators related to body fluids are TBW, ECF and ICF. The results of this experiment indicated that the TBW% on the first day of modeling was 60.42 ± 0.36 vs. 60.62 ± 1.97, the TBW of the model group was higher than that of the control group on the 9th days, and it was basically normal on the 18th day level (Fig. 1i). The content of ECF showed no difference (Fig. 1j).

### Ang II, AVP concentration and renin activity

As shown in **Supplemental Fig. 2**, the plasma concentration of Ang II in the modeling group gradually decreased with the prolongation of modelling time, and reached the lowest level on the 12th day. The concentration of Ang II in renal tissue on the 6th, 9th and 12th days was significantly lower than that in the control group (*p* < 0.05 or *p* < 0.01), and there was no difference in other time points. The trend of plasma renin activity was consistent with that of Ang II. With the prolongation of time, the renin activity decreased significantly, and it was 12.25 ± 2.06 vs. 167.93 ± 20.92 ng/ml.hr on the 12th day (*p* < 0.01). Plasma renin activity at day 18 was still lower than in the control group (21.03 ± 7.03 vs. 142.98 ± 31.79 ng/ml.hr, *p* < 0.05). Nevertheless, the renal tissue renin activity of the model group had no statistical significance compared with the control group at different time points. The results of plasma AVP indicated that the AVP concentration of the model group was significantly lower than that of the control group on day 6 (2.82 ± 0.28 vs. 8.65 ± 1.92 pmol/ml, *p* < 0.01), and then increased to slightly higher than that in the control group.

The results of correlation analysis showed that plasma AVP concentration was positively correlated with urinary sodium excretion, while plasma Ang II concentration and renin activity had a weak negative correlation with urinary sodium excretion (*p* > 0.05, **Supplemental Fig. 3**).

### Immunohistochemistry and Fluorescence

**Fig. 2:**
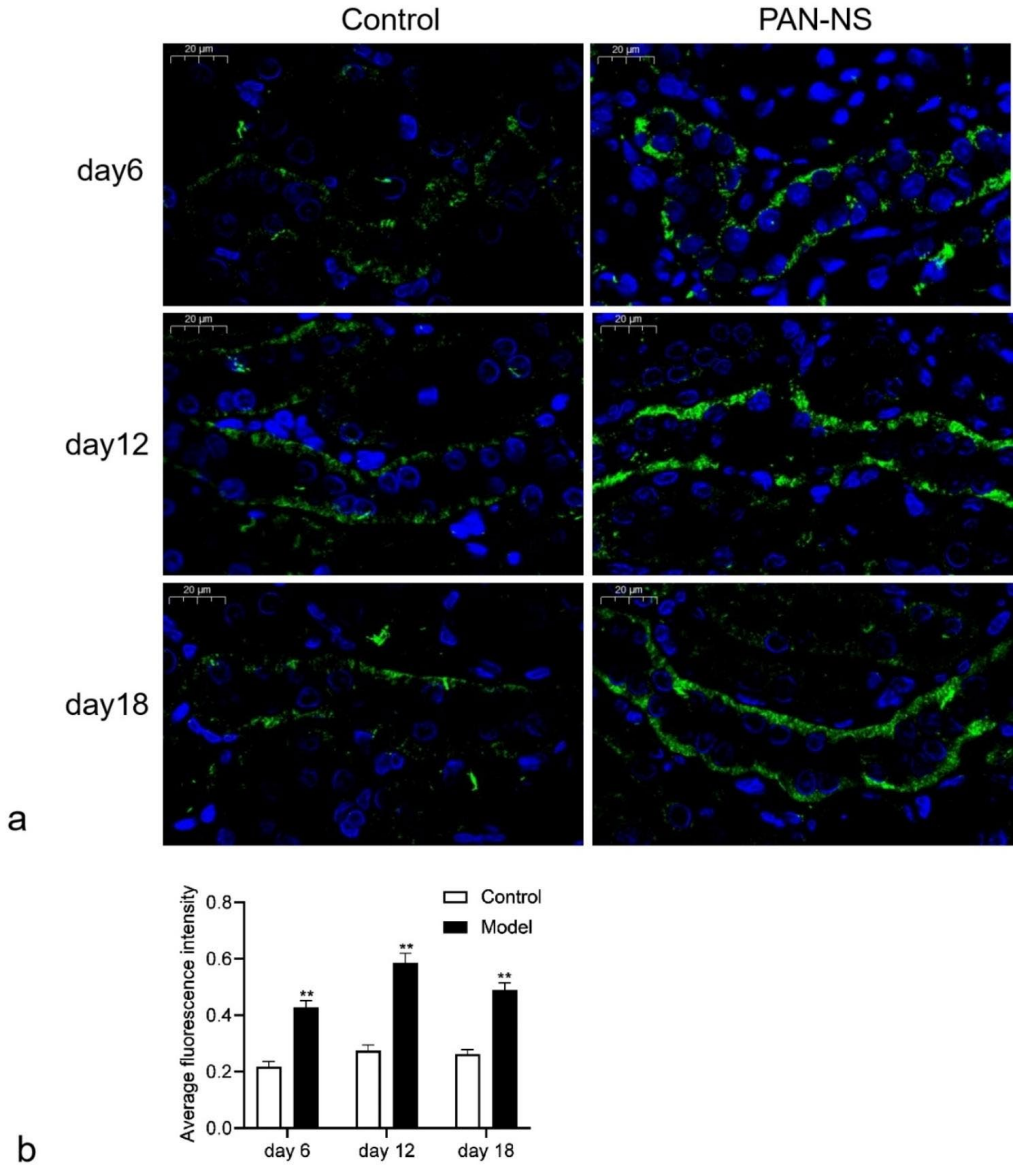
Immunofluorescence of γ-ENaC in CCD (×40, Scale bars, 20 μm). **a** Immunofluorescence microscopy of γ-ENaC in CCD after saline treatment or PAN treatment on day 6, 12 and 18. **b** Average fluorescence intensity value of γ-ENaC expression. ** indicates a statistical difference of *P <* 0.01 between the model group and the control group

Immunofluorescence results showed that γ-ENaC labeling was visible in the collecting ducts and connecting ducts of both control and model groups. In the cytoplasm of chief cells, γ-ENaC was diffusely distributed. Compared with the control group, there was no obvious apical targeting effect of γ-ENaC in the collecting ducts of rats in the model group (Fig. 2a). However, γ-ENaC expression was significantly increased in the model group compared with the control group at different time points (*p* < 0.01, Fig. 2b).

**Fig. 3:**
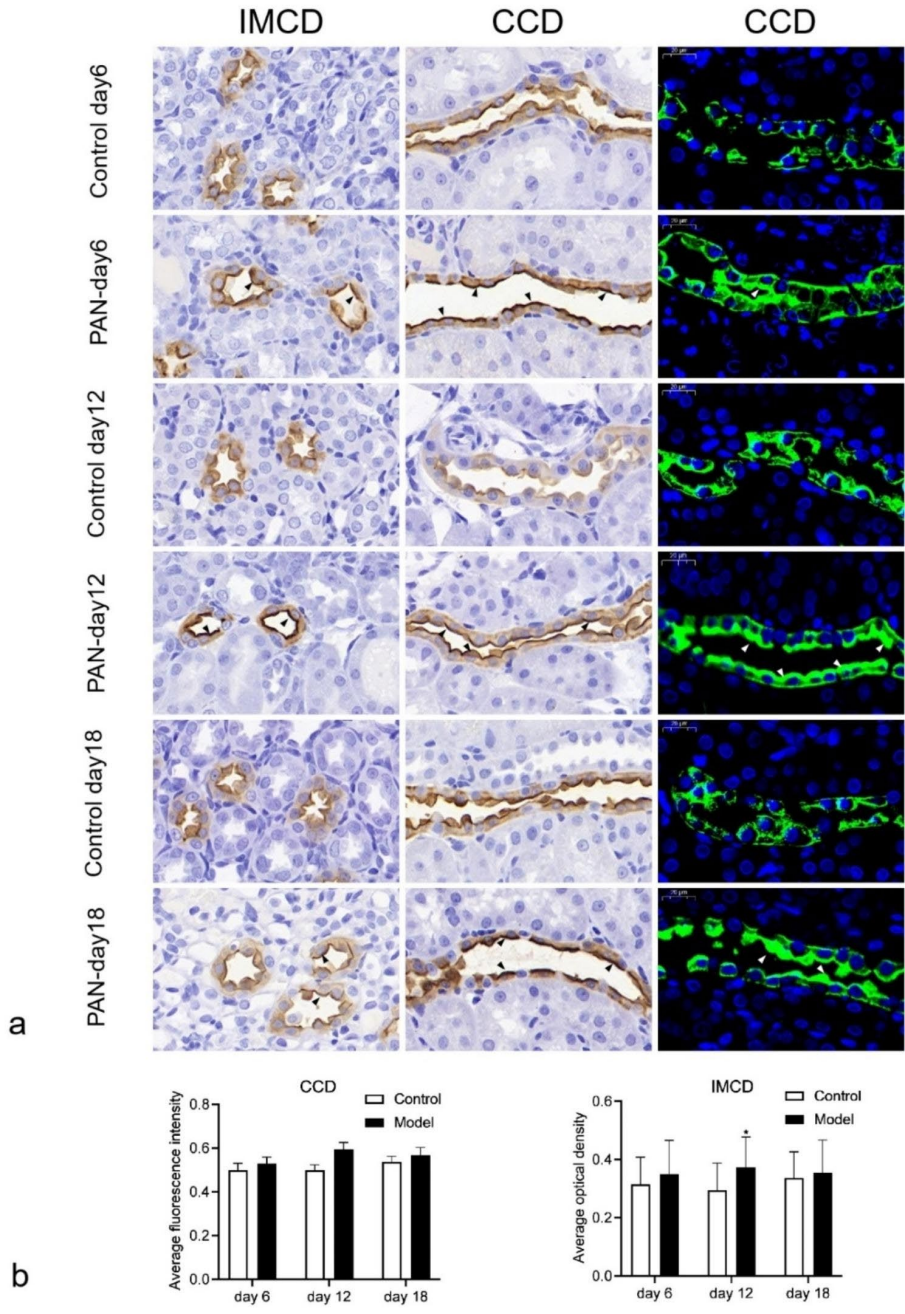
Immunohistochemistry and immunofluorescence of AQP2 in IMCD and CCD (×40, Scale bars, 20 μm). **a** Immunofluorescence microscopy of AQP2 in IMCD and CCD after saline treatment or PAN treatment on day 6, 12 and 18. **b** Average fluorescence intensity value of AQP2 expression in CCD. **c** Average optical density in IMCD. * indicates a statistical difference of *P <* 0.05 between the model group and the control group

The expression of AQP2 in the kidney is shown in Fig. 3. In control rats, AQP2 was mainly distributed evenly in the apical plasma membrane and basement membrane side of the cortical collecting duct and medullary collecting duct. At different modeling times in PAN-NS rats, the expression of AQP2 aggregated to the apical plasma membrane (Fig. 3a). Semi-quantitative results showed that AQP2 expression was significantly increased in inner medulla collecting ducts in PAN-NS rats only on day 12 (Fig. 3b and c).

### Western blot

**Fig. 4:**
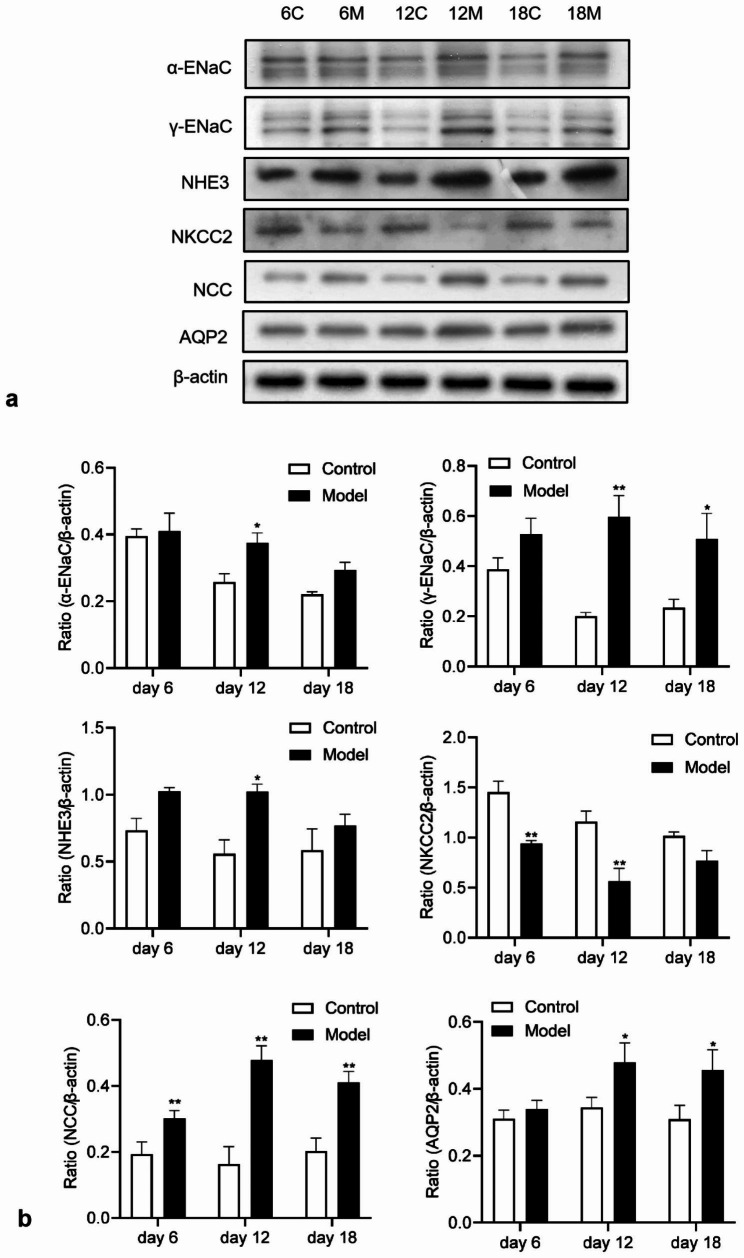
Changes of sodium transporters in renal cortex and AQP2 in renal medulla at different time points. **a** Electrophoretic blots for each protein **b** Expression level of sodium transporters in renal cortex and AQP2 in renal medulla. C stands for control group and M stands for model group, numerals represent different time points. Original blots are presented in Supplementary Fig. 4. * indicates a statistical difference of *P <* 0.05 between the model group and the control group, while ** indicates a statistical difference of *P <* 0.01 between the two groups

It can be seen from Fig. 4a that the expression of α-ENaC only on day 12 was significantly increased compared with the pair-fed control group (*p* < 0.05). The expression of γ-ENaC increased significantly on the 12th and 18th days (*p* < 0.05 or *p* < 0.01), and there was no significant difference in the changes on day 6. There was a significant increase in NHE3 on day 12, and there was no difference in change on day 18. NKCC2 was the only indicator of decreased expression among the tested proteins: A significant decrease began on the 6th day, reached the lowest value on the 12th day, and the expression was no different from the control group on the 18th day. At all-time points, NCC protein expression was significantly increased (*p* < 0.01), and the NCC expression in the control group was maintained at the same level. Notably, in pair-fed control rats, the expressions of α-ENaC, γ-ENaC and NKCC2 in the renal cortex all showed a downward trend, which may be related to the decrease in sodium intake.

The detection results of the intrarenal medulla (Fig. 4b) showed that the expression of AQP2 increased significantly on the 12th and 18th days (*p* < 0.05), and the expression of AQP2 in the pair-fed control group was relatively stable, indicating the intake of sodium content in the body may not affect changes in AQP2.

## Discussion

In this experiment, proteinuria began to appear on the 5th day after intraperitoneal injection of PAN, and the significant increase in urinary protein continued until 18 days, which is basically consistent with the literature reports [17]. It should be noted that urinary sodium excretion was greater on the first day of intraperitoneal injection of PAN, suggesting that urinary sodium excretion was excreted on the first day after PAN injection, may be due to the toxic effects of the drug on the renal tubules. In addition, the study noted a decrease in Na^+^-K^+^-ATPase activity in CCD on day 1 [8], at the peak of the urinary sodium excretion curve, urinary sodium excretion was reduced by approximately 75% in the PAN group. This part of the experimental study showed that after 12 days of PAN-NS, urinary sodium excretion was not different from the control group, which was consistent with the results reported in the literature for 18 days of PAN-NS [18]. Combined with the results of this experiment, we believe that the decrease in urinary sodium excretion is independent of the development of proteinuria and may be related to tubular damage rather than glomerular injury.

In part of the literature, researchers divided PAN-NS sodium retention and edema over time into two processes [15, 19]. The first course is marked by significant urinary sodium excretion with positive sodium balance and accumulation of ascites, and the second process is marked by significant improvement in ascites and persistent proteinuria. Our experimental results also support the phenomenon of different characteristics in different stages in the NS process, but based on the prolonged observation time of animals, on the basis of the decreased urinary sodium excretion stage and the urinary sodium excretion recovery stage, our dynamic observation indicates that urinary sodium excretion is stable at normal level from day 12 to day 18, but edema is still existed. We speculate that the body exhibits sodium retention in the early stage of NS, and then reaches a state of sodium balance, but it is still accompanied by water retention. Therefore, the pathogenesis of edema may be different in different disease processes, which may also be the reason for the poor efficacy of clinical natriuretic therapy for NS edema. It is of great significance to deeply explore the possible influencing factors and mechanisms of sodium-water retention in different stages of NS.

To explore the mechanism of NS edema, we first investigated the dynamic changes of peripheral circulation factors. The experimental results indicated that the plasma Ang II and renin activity levels of PAN-NS rats gradually decreased with the time course of modeling, which is consistent with NS patients exhibiting normal or lower plasma renin concentrations during periods of sodium retention [20]. However, reductions in Ang II levels and renin activity do not appear to be important mechanisms associated with PAN-NS sodium retention, the reasons are as follows: (1) The decrease of urinary sodium excretion in PAN-NS rats was earlier than that of Ang II level and renin activity; (2) No correlation between plasma Ang II level and renin activity and urinary sodium excretion was found throughout the experimental period. The decrease in plasma AVP concentration in PAN-NS was contrary to that reported in the literature. In vitro isolated and micro perfused rat CCD studies showed that AVP and cAMP mimics could stimulate sodium ion reabsorption in a short time; AVP and cAMP mimics increase ENaC apical targeting in A6 cells; high levels of plasma AVP have also been shown in PAN-NS rats [21–23]. However, PAN stimulation still induced sodium retention and ascites in AVP-knockout Brattleboro rats [8]. Therefore, it remains unclear whether peripheral circulatory factors are an important mechanism of sodium-water retention in NS. Since the decrease in blood volume can stimulate the release of AVP [24], whether the mechanism by which peripheral circulating factors are involved in the disease is related to blood volume remains to be further investigated.

In this experiment, significant sodium retention occurred on days 7 to 9 after modeling, during which the expression of different subtypes ENaC, NCC and NHE3 increased in renal cortex, while the expression of NKCC2 decreased in extrarenal medulla. Although no obvious increase in apical targeting of γ-ENaC was observed in this experiment, the protein expression of γ-ENaC was significantly increased, and the distribution of its apical plasma membrane was significantly increased relative to the control group, so the increase in γ-ENaC activity is still important mechanism of sodium retention in NS. In addition, Pendrin, as an important target for regulating Cl^−^/HCO_3_^−^, can also regulate the expression and activity of ENAC [25]. Sodium restriction in pendrin knockout mice showed a strong sodium excretion response, suggesting that the sodium transport mediated by ENaC in pendrin knockout mice is defective [26]. It may also reveal the regulatory role of ENaC and provide a basis for future research. Notably, the expression of NKCC2 decreased on day 18 of modeling, but the expression of other sodium transporters remained high, which is consistent with the generation of ascites. Studies have shown that NKCC2 activity and protein expression in cortex and medulla of PAN-NS rats are reduced [27, 28], our results also observed decreased expression of NKCC2 in renal cortical sites, possibly revealing a mechanism of feedback inhibition during NS edema. The results of western blotting indicated that the expression of NHE3 in the renal cortex of rats in the model group was higher than that in the control group at three time points, and the mechanism of its increased expression might be related to the activity of renal dopamine and its exchanger. Sampaio-Maia B et al. [13] showed that renal dopamine was decreased in PAN-NS rats, and renal dopamine could directly inhibit Na^+^-K^+^-ATPase activity or inhibit NHE3-mediated proximal tubular sodium reabsorption. Studies on NHE3 activity indicated that the Na^+^/H^+^ exchange activity corresponding to NHE3 antigen in nephrotic rats increased by 88%; a part of NHE3 existed in the active form of 9.6 S in the brush edge microvilli; the 2B9 antibody, which binds tightly to the active form of NHE3, resulted in an increased immune response to NHE3 [29, 30].

Studies have pointed out that the main factors affecting the body’s water reabsorption are the NaCl reabsorption in the ascending branch and the permeability of the collecting duct water [31]. In NS patients, urinary sodium excretion is normal at a certain stage, but AQP2 in the kidney and urine is still increased, suggesting that there is water retention in the body but not related to NaCl reabsorption [13, 14]. Therefore, the permeability of collecting duct water dominates the occurrence of edema at this stage. This process was consistent with the results of the present experiment, with urinary sodium excretion tending to balance on day 12, but AQP2 expression was elevated. AQP2 is an important member of the aquaporin family, regulated by the AVP V2 receptor, it increases collecting tube protein expression and apical targeting mediates water permeability through both short-term and long-term regulation [32]. AQP2 was abnormally increased in animal models of NS and was involved in body fluid retention [33]. However, the results of this experiment did not find the increase of AVP, on the contrary, AVP was significantly decreased in the early stage of modeling, which may reveal that AVP-independent mechanism regulates the expression of AQP2.

## Conclusions

Overall, we showed that intraperitoneal injection of PAN at 100 mg/kg induced marked NS features accompanied by edema. The occurrence of NS edema may be independent of the angiotensin system. The 18-day observation period can be divided into three stages: sodium retention, sodium compensation, and simple water retention. The mechanism is related to the increased expression of α-ENaC, γ-ENaC, NHE3 and NCC in a certain period of time, the compensatory decrease of NKCC2 expression and the continuous increase of AQP2 expression. Our study provides a scientific model carrier for the study of edema in NS, clarifies some mechanisms of the three stages of sodium and water retention, and provides some reference for subsequent studies on edema in NS.

### Electronic supplementary material

Below is the link to the electronic supplementary material.

Supplementary Material 1

## Data Availability

The datasets used and/or analyzed during the current study are available from the corresponding author on reasonable request.

## References

[1] Chen J, Qiao XH, Mao JH (2021). Immunopathogenesis of idiopathic Nephrotic Syndrome in children: two sides of the coin. World J Pediatr.

[2] Zabala Ramirez MJ, Stein EJ, Jain K (2023). Nephrotic Syndrome for the internist. Med Clin North Am.

[3] Hedin E, Bijelić V, Barrowman N, Geier P (2022). Furosemide and albumin for the treatment of nephrotic edema: a systematic review. Pediatr Nephrol.

[4] Nie X, Chanley MA, Pengal R, Thomas DB, Agrawal S, Smoyer WE (2018). Pharmacological and genetic inhibition of downstream targets of p38 MAPK in experimental Nephrotic Syndrome. Am J Physiol Renal Physiol.

[5] Pereira Wde F, Brito-Melo GE, de Almeida CA, Moreira LL, Cordeiro CW, Carvalho TG (2015). The experimental model of Nephrotic Syndrome induced by Doxorubicin in rodents: an update. Inflamm Res.

[6] Diamond JR, Bonventre JV, Karnovsky MJ. A role for oxygen free radicals in aminonucleoside nephrosis. Kidney Int 1986,29(2):478–83. 10.1038/ki.1986.24 3702206

[7] Fishman JA, Karnovsky MJ (1985). Effects of the aminonucleoside of puromycin on glomerular epithelial cells in vitro[J]. Am J Pathol.

[8] Deschênes G, Doucet A (2000). Collecting duct (Na^+^/K^+^)-ATPase activity is correlated with urinary sodium excretion in rat nephrotic syndromes. J Am Soc Nephrol.

[9] Yang M, Yue X, Zhou M, Liu H, Yao Y, Xuan Z, et al. Study on the effect of Danggui Shaoyao San on rats with Nephrotic Syndrome based on electrolytes. J Liaoning Univ Traditional Chin Med. 2017 Jan;19(01):39–41.

[10] Fan S, Ou M, Wang Y, Yang M, Xuan Z, Xu F (2021). The therapeutic effect of Danggui Shaoyao San and Astragalus membranaceus on adriamycin induced Nephrotic Syndrome in rats. J Hainan Med Coll.

[11] Xiao M, Bohnert BN, Grahammer F, Artunc F (2022). Rodent models to study sodium retention in experimental Nephrotic Syndrome. Acta Physiol (Oxf).

[12] Abdeen A, Sonoda H, Kaito A, Oshikawa-Hori S, Fujimoto N, Ikeda M (2020). Decreased excretion of urinary exosomal Aquaporin-2 in a puromycin Aminonucleoside-Induced Nephrotic Syndrome Model. Int J Mol Sci.

[13] Wang Y, Bu J, Zhang Q, Chen K, Zhang J, Bao X (2015). Expression pattern of aquaporins in patients with primary Nephrotic Syndrome with edema. Mol Med Rep.

[14] Li P. Analysis of clinicopathological characteristics and related factors of edema in patients with Nephrotic Syndrome. Fudan Univ, 2008.

[15] Sampaio-Maia B, Moreira-Rodrigues M, Serrão P, Pestana M (2006). Blunted renal dopaminergic system activity in puromycin aminonucleoside-induced Nephrotic Syndrome. Nephrol Dial Transplant.

[16] Hu L, Maslanik T, Zerebeckyj M, Plato CF (2012). Evaluation of bioimpedance spectroscopy for the measurement of body fluid compartment volumes in rats. J Pharmacol Toxicol Methods.

[17] Jo CH, Kim S, Kim GH (2020). Association of Proteinuria with urinary concentration defect in Puromycin Aminonucleoside Nephrosis. Electrolyte Blood Press.

[18] Cátia FC, Janete QS, Benedita SM, Liliana SS, Isabel SS, Roberto RA (2018). Calcitriol prevents Cardiovascular repercussions in Puromycin Aminonucleoside-Induced Nephrotic Syndrome. Biomed Res Int.

[19] Bae EH, Kim SW (2010). Changes in endothelin receptor type B and neuronal nitric oxide synthase in puromycin aminonucleoside-induced Nephrotic Syndrome. Korean J Physiol Pharmacol.

[20] Brown EA, Markandu ND, Roulston JE, Jones BE, Squires M, MacGregor GA (1982). Is the renin-angiotensin-aldosterone system involved in the sodium retention in the Nephrotic Syndrome?. Nephron.

[21] Pyo HJ, Summer SN, Niederberger M, Kim JK, Schrier RW (1995). Arginine vasopressin gene expression in rats with puromycin-induced Nephrotic Syndrome. Am J Kidney Dis.

[22] Hawk CT, Li L, Schafer JA (1996). AVP and aldosterone at physiological concentrations have synergistic effects on Na^+^ transport in rat CCD. Kidney Int Suppl.

[23] Kleyman TR, Ernst SA, Coupaye-Gerard B (1994). Arginine vasopressin and forskolin regulate apical cell surface expression of epithelial Na^+^ channels in A6 cells. Am J Physiol.

[24] Bankir L, Bichet DG, Morgenthaler NG (2017). Vasopressin: physiology, assessment and osmosensation. J Intern Med.

[25] Soleimani M (2015). The multiple roles of pendrin in the kidney. Nephrol Dial Transplant.

[26] Kim YH, Pech V, Spencer KB, Beierwaltes WH, Everett LA, Green ED (2007). Reduced ENaC protein abundance contributes to the lower blood pressure observed in pendrin-null mice. Am JPhysiol Renal Physiol.

[27] Kim SW, de Seigneux S, Sassen MC, Lee J, Kim J, Knepper MA (2006). Increased apical targeting of renal ENaC subunits and decreased expression of 11betaHSD2 in HgCl2-induced Nephrotic Syndrome in rats. Am J Physiol Renal Physiol.

[28] Bou Matar RN, Malik B, Wang XH, Martin CF, Eaton DC, Sands JM (2012). Protein abundance of urea transporters and aquaporin 2 change differently in nephrotic pair-fed vs. non-pair-fed rats. Am J Physiol Renal Physiol.

[29] Ambühl P, Amemiya M, Preisig PA, Moe OW, Alpern RJ (1998). Chronic hyperosmolality increases NHE3 activity in OKP cells. J Clin Invest.

[30] Besse-Eschmann V, Klisic J, Nief V, Le Hir M, Kaissling B, Ambühl PM (2002). Regulation of the proximal tubular sodium/proton exchanger NHE3 in rats with puromycin aminonucleoside (PAN)-induced Nephrotic Syndrome. J Am Soc Nephrol.

[31] Sonntag SR, Ziemens A, Wulfmeyer VC, Milatz S, Bleich M, Himmerkus N (2018). Diuretic state affects ascending thin limb tight junctions. Am J Physiol Renal Physiol.

[32] Olesen ET, Fenton RA (2017). Aquaporin-2 membrane targeting: still a conundrum. Am J Physiol Renal Physiol.

[33] Liang CL, Zhang PC, Wu JB, Liu BH, Yu-He, Lu RR (2019). Zhen-Wu-Tang attenuates adriamycin-induced Nephropathy via regulating AQP2 and miR-92b. Biomed Pharmacother.

