# Supplementary material for: Characteristics of sodium and water retention in rats with nephrotic syndrome induced by puromycin aminonucleoside
Source: BMC Nephrol. 2023 Oct 25;24:309. doi: 10.1186/s12882-023-03367-z (PMC10599035; doi:10.1186/s12882-023-03367-z)
Supplement: Supplementary file 1 — Supplementary Material 1 [file 12882_2023_3367_MOESM1_ESM.docx]

***Characteristics of sodium and water retention in rats with nephrotic syndrome induced by puromycin aminonucleoside***

Zaiping Xu^a^, Yunlai Wang^a, b, c*^, Ye Feng^a^, Mo Yang^d^, Gaoxiang Shi^e^, Zihua Xuan^a^, Fan Xu^a, b, c*^

^a^ School of Pharmacy, Anhui University of Chinese Medicine, Hefei, Anhui, China

^b^ Anhui Province Key Laboratory of Chinese Medicinal Formula, Hefei, Anhui, China

^c^ Institute for Pharmacodynamics and Safety Evaluation of Chinese Medicine, Anhui Academy of Chinese Medicine, Hefei, Anhui, China

^d^ Scientific Research and Technology Center, Anhui University of Chinese Medicine, Hefei, Anhui, China

^e^ School of Integrated Chinese and Western Medicine, Anhui University of Chinese Medicine, Hefei, Anhui, China

Corresponding Author:

Yunlai Wang

School of Pharmacy

Anhui University of Chinese Medicine

Longzihu Road 350

Hefei, Anhui, 230012, China

Tel:15209880323

E-mail:ylwang@ahtcm.edu.cn

Fan Xu

School of Pharmacy

Anhui University of Chinese Medicine

Longzihu Road 350

Hefei, Anhui, 230012, China

Tel:13505514407

### E-mail:xufanahcm@qq.com

### Supplementary Material


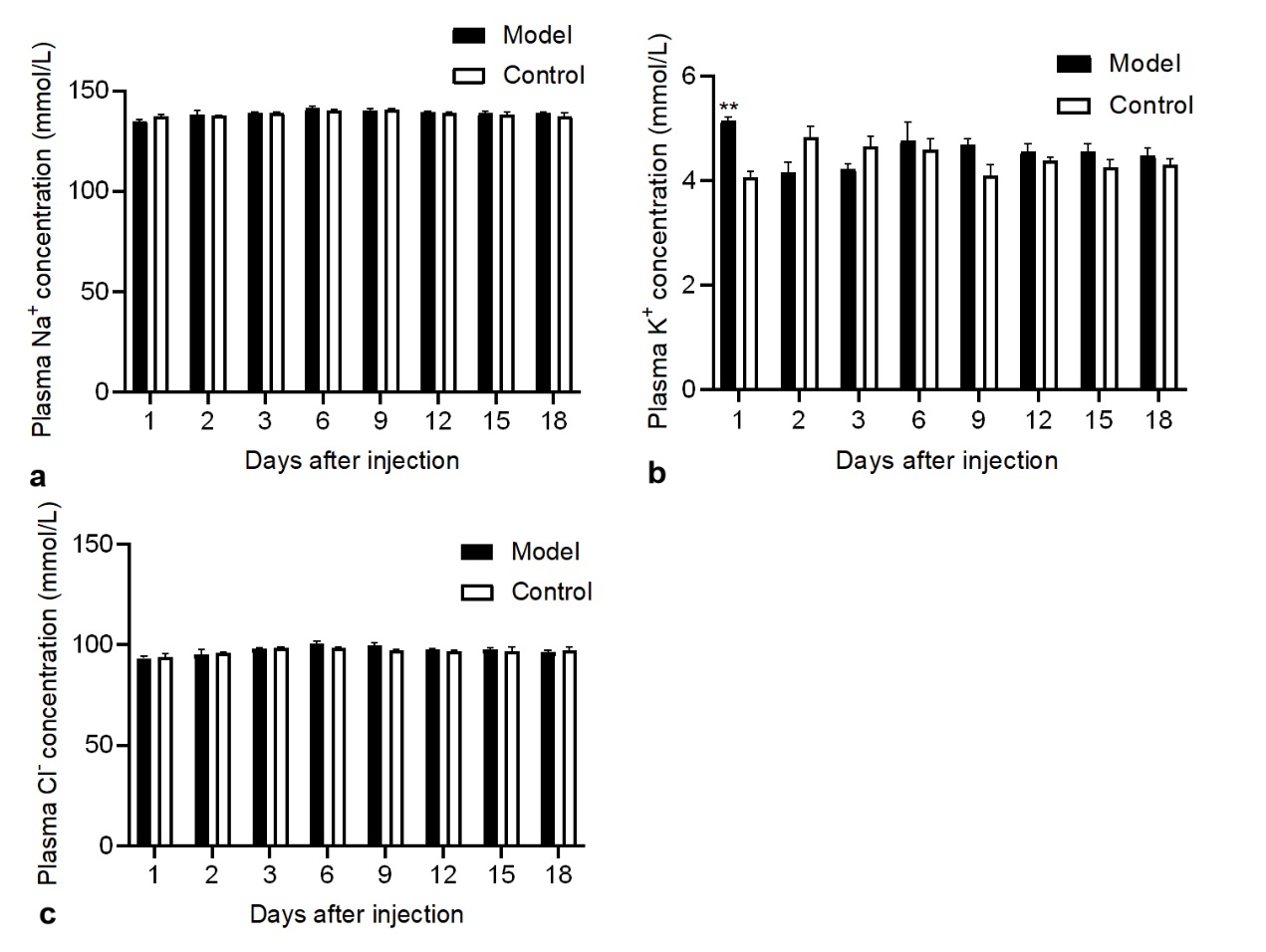


Supplemental Fig. 1. Changes of plasma electrolytes in PAN and control rats throughout the study. **a** The time courses of plasma Na^+^ concentration. **b** The time courses of plasma K^+^ concentration. **c** The time courses of plasma Cl^-^ concentration. The values are mean + SE from four animals. ** indicates a statistical difference of *P*<0.01 between the model group and the control group.


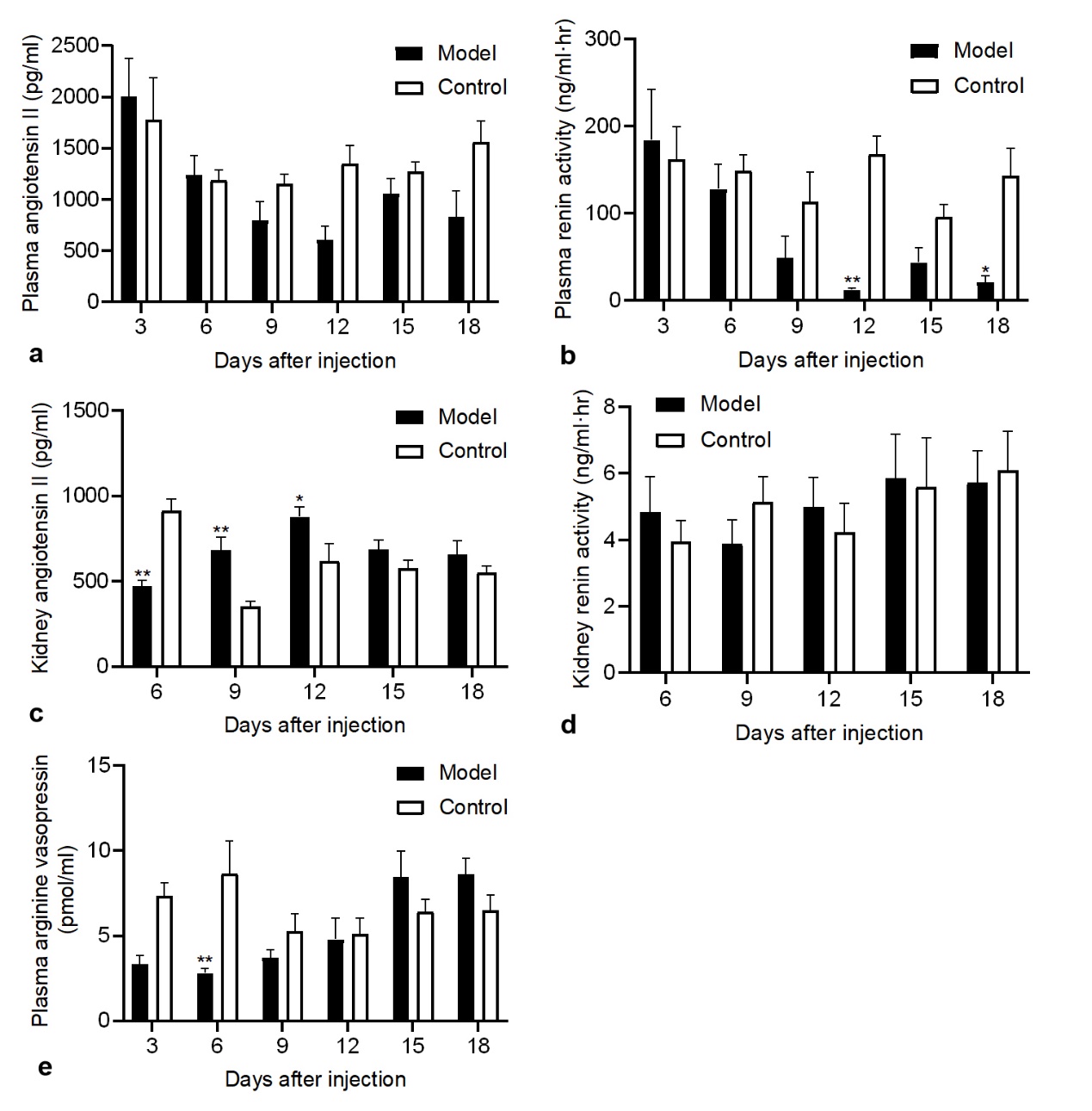


Supplemental Fig. 2. Changes of peripheral factor in PAN and control rats throughout the study. **a** Angiotensin II concentration in plasma. **b** Renin activity in plasma. **c** Angiotensin II concentration in kidney tissue. **d** Renin activity in kidney tissue. **e** AVP concentration in plasma. The values are mean + SE from four animals. * indicates a statistical difference of *P*<0.05 between the model group and the control group, while ** indicates a statistical difference of *P*<0.01 between the two groups.


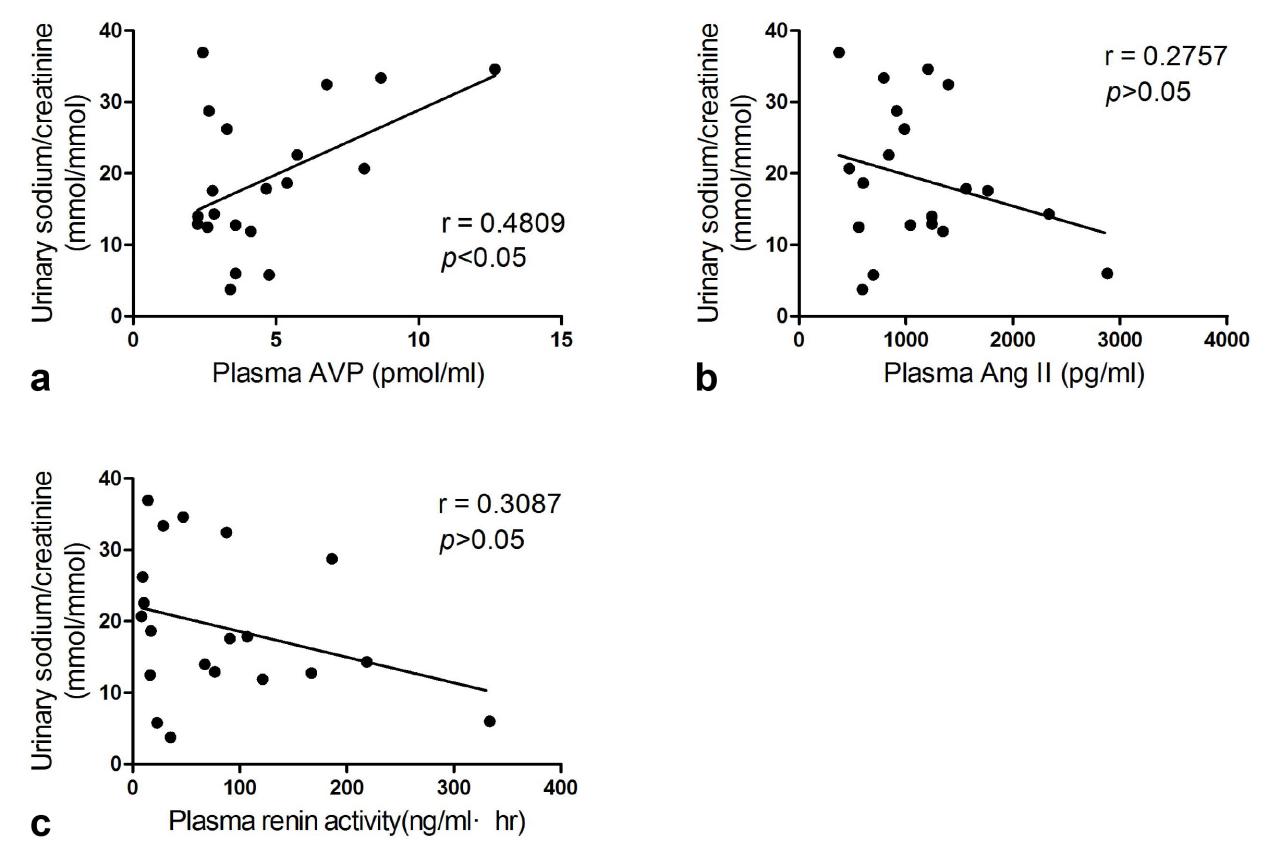


Supplemental Fig. 3. Correlation of urinary sodium with peripheral factors. **a** Correlation between urinary sodium and Ang II. **b** Correlation between urinary sodium and AVP. **c** Correlation between urinary sodium and renin activity. Linear regression analysis was performed by Pearson correlation.


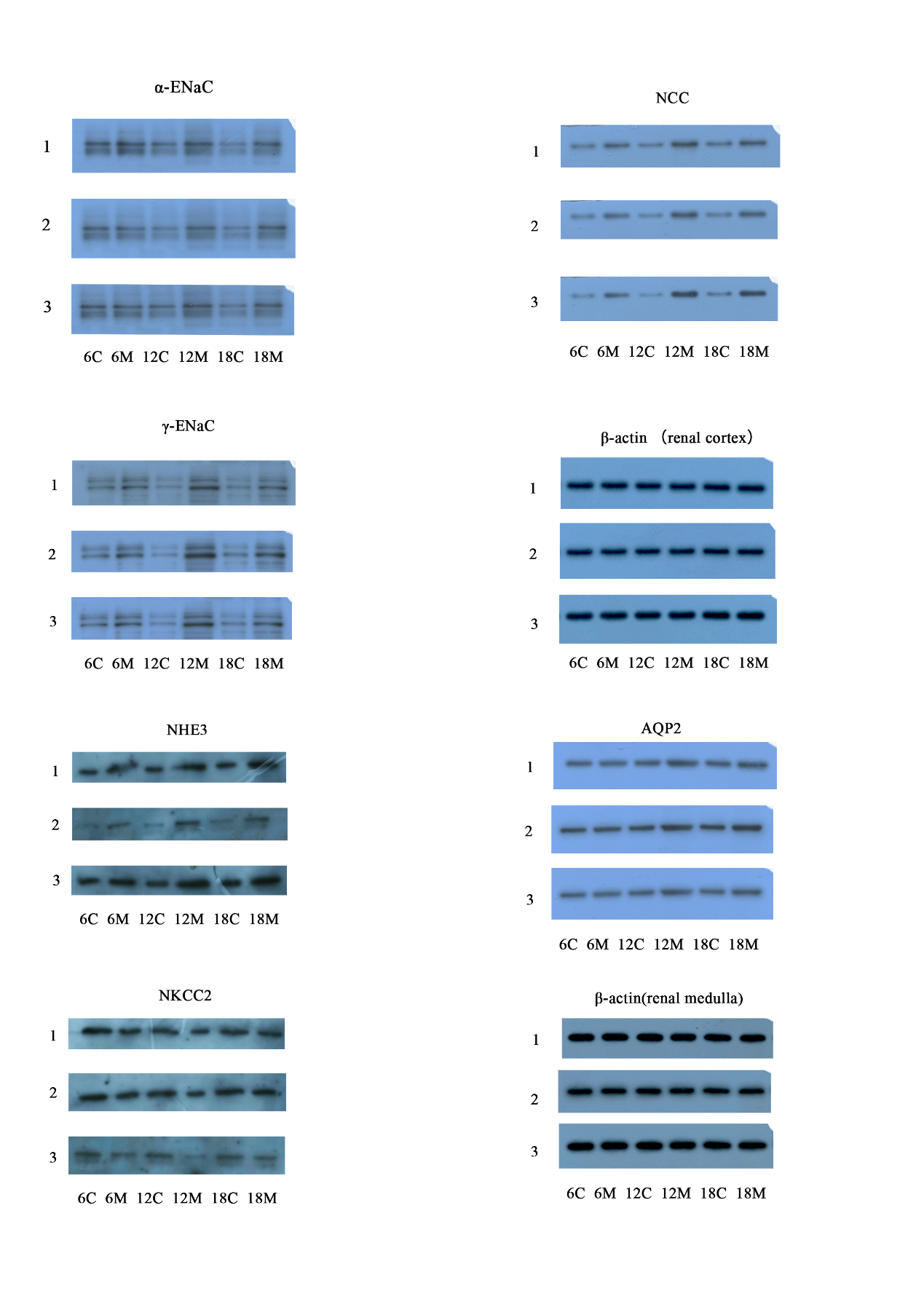


Supplemental Fig. 4. Original blots.
